# Application of Mesoporous Silica Nanoparticle-Chitosan-Loaded BMP-2 in the Repair of Bone Defect in Chronic Osteomyelitis

**DOI:** 10.1155/2022/4450196

**Published:** 2022-07-31

**Authors:** Min Yi, Yu Nie, Chengyun Zhang, Bin Shen

**Affiliations:** ^1^Department of Orthopedics, Orthopedic Research Institute, West China Hospital, Sichuan University, Chengdu, 610041 Sichuan, China; ^2^Engineering Research Center in Biomaterials, Sichuan University, Chengdu, 610041 Sichuan, China

## Abstract

In order to test the effectiveness of nanoparticle- (NP-) loaded bone morphogenetic protein 2 (BMP-2) in chronic osteomyelitis (CO) complicated with bone defect, a new nanodrug delivery system composed of mesoporous silica NP (MSN) and chitosan were used to load BMP-2 and transfer it to the target region. Bone marrow mesenchymal stem cells (BMSCs) were purchased and cultivated to detect the osteogenesis of chitosan-MSN (Chi-MSN) and polylactic acid glycolic acid (PLGA) delivery system. In addition, the osteogenesis of Chi-MSN was further determined by constructing a bone defect mouse model. In physicochemical property test, we found Chi-MSN NPs could effectively maintain stability in vivo and had pH response characteristics. As a result, the release efficiency of dexamethasone (Dex) and BMP-2 in the environment with pH 7.4 was less, while it increased significantly in pH 6, so as to reduce the BMP-2 and Dex loss during transportation in vivo. Otherwise, we found that the permeation efficiency of Chi-MSN was significantly higher than that of PLGA delivery system, so as to effectively transport BMP-2 and Dex to action target. In the BMSC test, we found that Chi-MSN could better promote their activity and osteogenesis, and the expression of osteogenesis-related genes (runt-related transcription factor 2 (RUNX-2), osteopontine (OPN), alkaline phosphatase (ALP), and osteopontine (OCN)) in the Chi-MSN group was higher. In the bone defect mouse model test, we also found obviously increased bone trabecula number and thickness by Chi-MSN, contributing to better repair of bone defects. Therefore, BMP-2@Chi-MSN may be a better choice for the therapy of CO complicated with bone defect in the future.

## 1. Introduction

In recent years, the number of patients with multiple injuries has increased year by year due to the frequent occurrence of traffic accidents and falling injuries, and most of them are complicated with open limb fractures. Because these patients' wounds are often polluted, with less surface tissue coverage and relatively poor blood supply, they are prone to delayed healing in the later stage and may turn into chronic osteomyelitis (CO) [1, 2], which will greatly affect patients' life quality. For CO patients' therapy, it is necessary to clear dead bone, close dead cavity, reconstruct bone defect, and restore blood supply [3, 4]. In terms of bone defect reconstruction, autologous bone or allogeneic bone can be selected for filling. Although autologous bone filling has good compatibility, it is easy to cause damage to the bone removal site. Otherwise, allogeneic bone may cause rejection and infectious diseases [5]. Therefore, the reform and improvement of bone defect repair technology is an important link in CO therapy. With the development of tissue bioengineering technology, it has been more and more used in clinical therapy and brought a variety of technological innovations, including bone defect repair. For example, the combination of bone marrow mesenchymal stem cells (BMSCs) with scaffold materials and related biological factors to reconstruct bone defect through bone conduction and bone induction provides a new choice for CO therapy.

BMSCs have strong self-renewal and repair ability and can differentiate into a variety of cells, including chondrocytes and osteoblasts [6]. Therefore, BMSCs are commonly used seed cells in the process of bone repair, and how to promote the differentiation of BMSCs into osteoblasts and promote their maturation and proliferation is an important part of repairing defective bone tissue [7]. Playing a vital part in bone growth and formation, bone morphogenetic protein 2 (BMP-2) can promote the expression of specific functional proteins in BMSCs through SMAD signal transduction pathway to exert the effect of bone induction [8, 9]. BMP-2 is essential for osteogenesis and is the starting point of the whole process [10]. In addition, relevant studies show that dexamethasone (Dex) also has the effect of inducing BMSCs to differentiate into osteoblasts and can improve the activity of osteoblasts [11], so as to strengthen the bone induction of BMP-2. Furthermore, the inflammatory state of CO will stimulate osteoclasts and increase their activity, so as to inhibit the formation of new bone. As Dex can inhibit the release of inflammatory factors in vivo, it can indirectly reduce osteoclast activity [12]. Therefore, the combination of BMP-2 and Dex will play a complementary effect in bone defects repair of CO.

In the process of bone repair, the delivery and release of BMP-2 and Dex are also the key point to determine clinical efficacy. However, developing new reliable drugs is costly and time-consuming, often involving high-throughput screening (HTS) and requiring complex in vivo and in vitro validations [13]. Current researchers are paying attention to how to use carriers or drug carriers to achieve efficient drug delivery systems to produce customized drugs which can control the dosage and release rate of the drug [14]. In order to avoid the premature release of Dex and BMP-2, so as to reduce the loss during transportation, mesoporous silica nanoparticle (MSN) was selected as an effective carrier. MSN has good biocompatibility with the body and is a drug carrier widely used in clinical research [15]. The large space structure inside MSN can be used to load a variety of drugs, and its adjustable hole structure is conducive to controlling the absorption and release of drugs [16]. Otherwise, the surface of MSN can be modified with a variety of functional groups, so as to improve the transmission rate of MSN delivery system in vivo and reduce drug loss, thus improving drug utilization efficiency [17, 18].

In this study, we modified chitosan to make MSN have the pH response, so that it can react in acidic environment, which can effectively reduce drug loss during transportation in vivo [19]. Otherwise, chitosan has the functions of antibacterial and immune regulation and can stimulate the conduction function of osteoblasts [20, 21], which can further enhance the efficacy of MSN delivery system in bone defect therapy of CO. Therefore, we used chitosan-MSN (Chi-MSN) delivery system to deliver Dex and BMP-2 in this study and explored their effects in bone defect repair of CO. The conclusions are as follows.

## 2. Materials and Methods

### 2.1. The Preparation of BMP-2@Chi-MSN Nanodelivery System

#### 2.1.1. The Synthesis of MSN

NaOH solution was added to 500 mL dH2O to adjust the pH to 11, in which 560 mg hexadecyltrimethylammonium bromide (CTAB) supplied by Sigma-Aldrich, St. Louis, MO, USA (H5882-100G) was dissolved. Following 15 min of stirring of the above mixture (50°C), 2.6-g tetraethyl orthosilicate (TEOS) supplied by Sigma-Aldrich (333859-25ML) was added. This was followed by mixture stirring (2 h) and supernatant removal by centrifugation. The obtained precipitate was then cleaned twice with dH2O and alcohol. The resulting particles were subjected to continuous thermal degradation treatment (550°C, 4 h) to remove CTAB. Eventually, MSN was obtained by vacuum drying.

#### 2.1.2. Dex and BMP-2 Loading into Chitosan-Modified MSN

**Figure 1:**
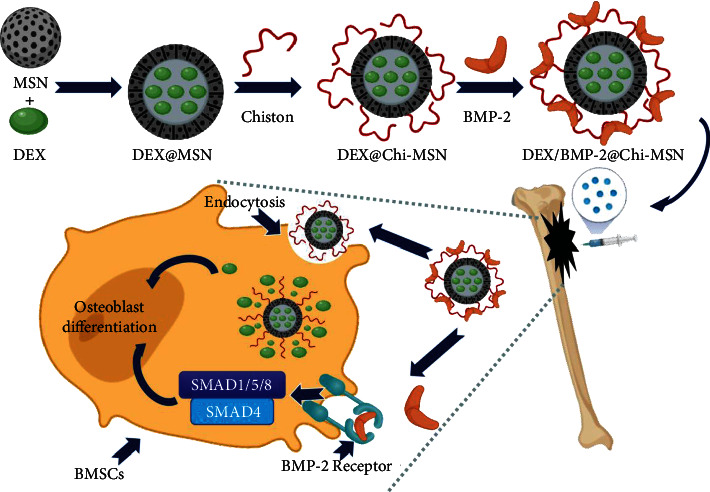
Schematic diagram of assembly process and mechanism of BMP-2@Chi-MSN.

0.3 g MSN was dissolved in 5 ml absolute ethanol, into which 50 mg Dex was placed. The above mixture was stored in a 26°C oscillator after 5 minutes of emulsification. After 4 hours, 25 mL chitosan/glycidoxypropyltrimethoxysilane solution (40 : 1) (440167, Merck, Darmstadt, Germany) was added in the above mixture. After 8-hour reaction at 26°C, the mixed solution was filtered and washed with dH2O to obtain Dex@Chi-MSN. Then, BMP-2 solution (ab80798, Abcam, USA) was added in Dex@Chi-MSN solution, and after soaking for 4 hours, the solution was filtered again to obtain BMP-2@Chi-MSN (Figure 1). Eventually, BMP-2@Chi-MSN was lyophilized and stored at 4°C for standby.

At the same time, we assemble poly (lactic co glycolic acid) (PLGA) with Dex and BMP-2 based on the conventional protocol to form BMP-2@PLGA delivery system as control.

### 2.2. Cell Culture

Among osteogenic stem cells, BMSCs belong to an important type that can differentiate into osteoblasts and chondrocytes to repair bone damage in vivo [22]. In this study, mouse BMSCs (CP-M131, Procell, Wuhan, China) planted in the wells of 24-well plates were immersed in a mouse BMSC-specific medium (CM-M131, Procell) that was placed in a 5% CO2 and 37°C cell incubator (51032124, Thermo Fisher Scientific, Waltham, MA, USA) for 24 h. The cultured cells were then diluted to a concentration of 4 × 104/mL for standby.

### 2.3. Establishment of Bone Defect Mouse Model

Nine male B6/J mice (Beijing Huafukang Biotechnology Co., Ltd, China) of eight weeks old and 100 g–150 g weight were selected to construct bone defect animal model. After the mice were anesthetized with ketamine (150 mg/kg), a 2 cm long incision was made at the right thigh, and the subcutaneous tissue was separated layer by layer to fully expose the femur. Then, the same high-pressure sterilized drill bit was used to drill a hole at the epiphysis of the femoral shaft, resulting in bone defect. Following bone wax filling of the lesion, all the postsurgery mice were placed on a heating pad until they regained mobility, and then, bone defect mouse models were successfully established. The above mice were divided in three groups averagely. Then, saline, BMP-2@PLAG, and BMP-2@Chi-MSN were injected into the lesion site of the modeled mice, respectively. The injection was performed once every three days for 14 days. This study was ratified by the Animal Care and Treatment Committee, and all experiments were carried out in compliance with laboratory animal care guidelines to minimize animal stress/distress.

### 2.4. Physicochemical Property Test of BMP-2@Chi-MSN

#### 2.4.1. Stability Test

In order to test the stability of Chi-MSN nanodelivery system, so as to evaluate whether it can effectively transport BMP-2 to the target, we observed its size change in PBS and PBS+10% serum, which was used to simulate the blood environment in vivo. The sizes of Chi-MSN drug delivery system were observed by scanning electron microscopy (SP8, Leica, Wetzlar, Germany).

#### 2.4.2. pH Response Characteristic Test

During transportation in vivo, the early release of drugs will not only cause drug loss and reduce the use efficiency but also cause adverse reactions. As the pH in vivo is about 7.35–7.45, detecting the pH sensitivity of nanodelivery system is important in judging the drug loss rate during delivery process. Different proportion of NaHCO3 and HEPES buffer reagent were added into saline to adjust pH value to 7.4 and 6, and the above configured solution would be used to test the release efficiency of Dex and BMP-2 in different delivery systems. 20 mg BMP-2@Chi-MSN and BMP-2@PLGA were added in the above solution at 37°C. For Dex release test, the above mixture was centrifuged at 1000 rpm to collect supernatant, whose Dex concentration was then quantified using high-performance liquid chromatography. For BMP-2 release test, the above mixture was centrifuged at 1000 rpm for supernatant acquisition. Then, the supernatant was detected by BMP-2 ELISA kit (ml002216, Shanghai enzyme-linked Biotechnology Co., Ltd, China) for BMP-2 concentration. The remaining sediment would be mixed with dH2O for the next test.

#### 2.4.3. Permeability Test

Before testing the therapeutic effects of BMP-2@Chi-MSN, we used PLGA and Chi-MSN coupled with Alexa Fluor®405 to test its capacity to infiltrate into the target tissue. Male B6/J mice (8-week old) were chosen as research object and divided into PLGA and Chi-MSN group for PLGA and Chi-MSN solution injection into the femoral marrow cavity, respectively. Every 12 hours, one mouse in each group was taken out and the injection site was dissected to observe the cells labeled by Alexa Fluor®405 with confocal microscopy (FV3000, OLYMPUS, Japan). The greater the number of labeled cells and the brighter the color, the stronger the infiltration capacity.

### 2.5. Cell Activity and Apoptosis Detection

The proliferation activity and apoptosis of BMSCs can be used to evaluate osteogenic effect of nanodelivery system. 1 mL BMSC diluent prepared in 2.2 was added in 12-well plates and divided into control group (without any treatment), PLGA group (add BMP-2@PLGA), and Chi-MSN group (add BMP-2@Chi-MSN). Then, the above cells were cultured in mouse BMSC-specific medium for another 24 h in cell incubator. BMSC activity and apoptosis detection would be performed by immunofluorescence (IF) at 12th and 24th hour. Anti-ki67 and anti-caspase 3 (ab15580, ab32351, Abcam, USA) were used for the labeling of active cells and apoptotic BMSCs in each group, respectively. Cell viability observation was carried out using a confocal microscope.

### 2.6. BMSC Mineralization and Osteogenesis Detection

The mineralization level of BMSCs is an important index to evaluate its ability to repair bone defects, and we evaluated it by alkaline phosphatase (ALP). As shown in 2.5, BMSC diluent will be assigned to control, PLGA, and Chi-MSN groups, and AP-TNAP ALPL Polyclonal Antibody Cy3 Conjugated (C03321Cy3, Nanjing Chuanbo Biotechnology Co., Ltd, China) was used to stain and detect with IF at 4th, 7th, and 14th day after culture. Otherwise, osteocalcin (OCN), runt-related transcription factor 2 (RUNX-2), and osteopontin (OPN) were specific osteoblast genes related to BMP-2 signaling pathway. Alexa Fluor® 647 Anti-RUNX2 antibody (ab215955, Abcam), OCN Antibody FITC Conjugated (C05448F, Nanjing Chuanbo Biotechnology Co., Ltd), and Alexa Fluor® 488 Anti-OPN antibody (ab282004, Abcam) were used to stain and detected with IF at 4th and 7th day after culture.

In the quantitative detection of ALP, RUNX-2, OCN, and OPN, the relative mRNA expression of above indicators in each group was detected by PCR. TRIzol reagent (Sigma) isolated total RNA. cDNA was synthesized using the M-MLV reverse transcription kit (Promega) as instructed the manufacturer's recommendations. After that, ChamQ Universal SYBR qPCR Master Mix (Vazyme Biotech, Nanjing, China) and QuantStudio 3 instrument (Thermo Fisher Scientific) were used for qPCR. The mRNA level of the GAPDH housekeeping gene served as a control, and the levels of genes were computed via the 2^-*ΔΔ*C^t method (see Table 1 for the primer information).

### 2.7. Therapy Effect Evaluation of BMP-2@Chi-MSN

Four weeks after saline, PLGA, or Chi-MSN injection, the mice were killed to collect the injected lesions. First, the macroscopic images, including bleeding, swelling, and inflammatory looking, were observed. The lesions were prepared as slices for HE staining, after which microscopical evaluation of trabecular number and thickness was performed. Each index was calculated and recorded after averaging the data obtained in 3 high-power fields (HPF, 40x).

### 2.8. Statistical Processing

GraphPad Prism 8.0 (GraphPad Software, La Jolla, CA, USA) analyzed and visualized the data. Data were presented as the mean ± SEM. Each test run at least in triplicate unless otherwise specified. Two-tailed unpaired *t*-test was utilized to identify significant differences between the mean values of groups. Differences between groups were determined using two-way ANOVA. *P* < 0.05 was the level of significance.

## 3. Results and Discussion

### 3.1. Characteristics of BMP-2@Chi-MSN Delivery System

**Figure 2:**
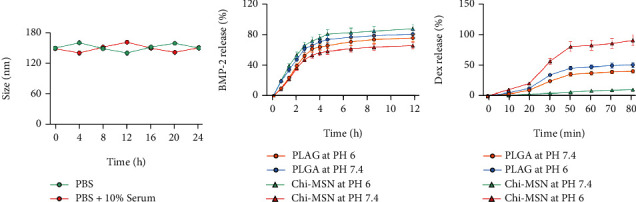
Physicochemical characterization of BMP-2@Chi-MSN. (a) Stability test of BMP-2@Chi-MSN at varied stored medium. (b) BMP-2 release rate of PLAG and Chi-MSN at different pH. (c) Dex release rate of PLAG and Chi-MSN at different pH.

The stability of delivery system is important in reducing drug loss and adverse reactions [23]. We found Chi-MSN delivery system could maintain similar size in PBS and serum (Figure 2(a)). Otherwise, compared with PLGA delivery system, Chi-MSN delivery system had obvious pH response characteristics. In the environment with pH 6, more Dex and BMP-2 were released, especially Dex (Figures 2(b) and 2(c)), suggesting that the release efficiency of Chi-MSN was significantly improved in the acidic environment. The above results suggested that Chi-MSN could effectively remain stable and reduce drug release during delivery process in vivo, so as to improve drug treatment efficiency.

### 3.2. Chi-MSN Delivery System Can Better Penetrate into Bone Tissue

**Figure 3:**
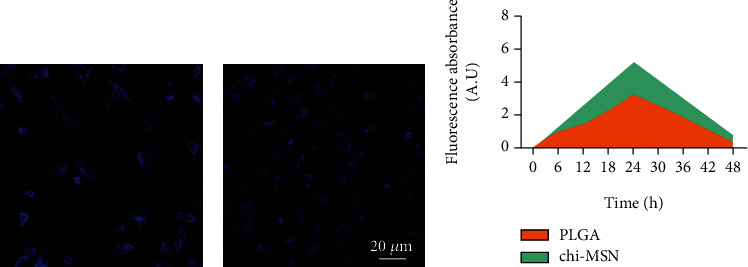
Infiltration ability of BMP-2@Chi-MSN in bone marrow tissue. (a) Infiltration ability of PLGA in bone marrow tissue. (b) Infiltration ability of Chi-MSN in bone marrow tissue. (c) Quantitative fluorescence evaluation in bone tissue after adding fluorescein-coupled PLGA and Chi-MSN.

The good permeability of delivery system at the target is the key to improve clinical efficacy [24]. Compared with PLGA, Chi-MSN could better penetrate into bone tissue cells and had higher fluorescence intensity in bone tissue (Figure 3). Therefore, Chi-MSN can better play an osteogenic role in BMSCs, which can promote the differentiation and maturation of BMSCs, thus better repairing bone defects.

### 3.3. Chi-MSN Delivery System Can Effectively Improve BMSC Activity

**Figure 4:**
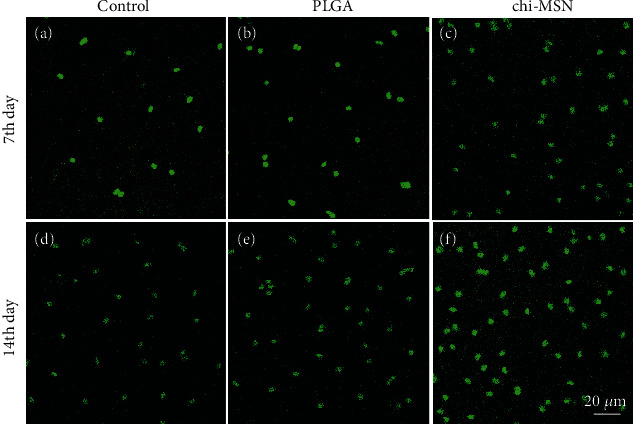
BMSC activity evaluation. (a–c) Anti-Ki67 expression in control, PLGA, and Chi-MSN groups at 7th day. (d–f) Anti-Ki67 expression in control, PLGA, and Chi-MSN groups at 14th day.

BMSC activity is of great significance for the improvement of their differentiation ability and osteogenic function [25]. The fluorescence intensity of BMSCs treated with PLGA and Chi-MSN was found to be markedly higher compared with the control group, especially in the Chi-MSN group (Figure 4), suggesting that Chi-MSN could better promote BMSC proliferation.

### 3.4. Chi-MSN Delivery System Can Effectively Inhibit BMSC Apoptosis

**Figure 5:**
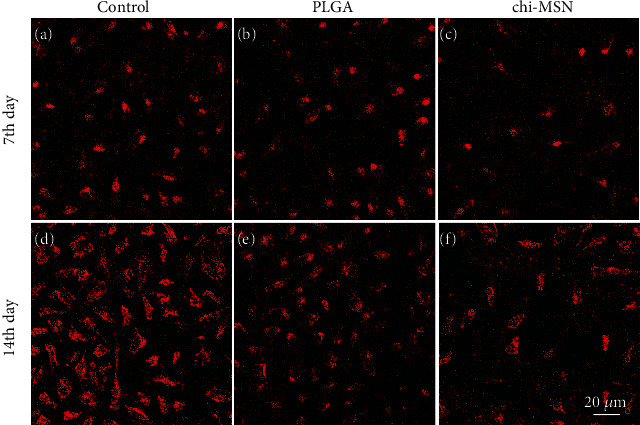
BMSC apoptosis detection. (a–c) Anti-caspase 3 expression in control, PLGA, and Chi-MSN groups at 7th day. (d–f) Anti-caspase 3 expression in control, PLGA, and Chi-MSN groups at 14th day.

Apoptosis degree of BMSCs will influence the osteogenic function of Chi-MSN delivery system and can reflect their proliferative activity from the side. It was found that compared with the control group, the fluorescence intensity in PLGA and Chi-MSN group was higher, especially in the Chi-MSN group (Figure 5), suggesting that Chi-MSN could better inhibit BMSC apoptosis.

### 3.5. Chi-MSN Delivery System Can Effectively Improve the Mineralization Ability of BMSCs

**Figure 6:**
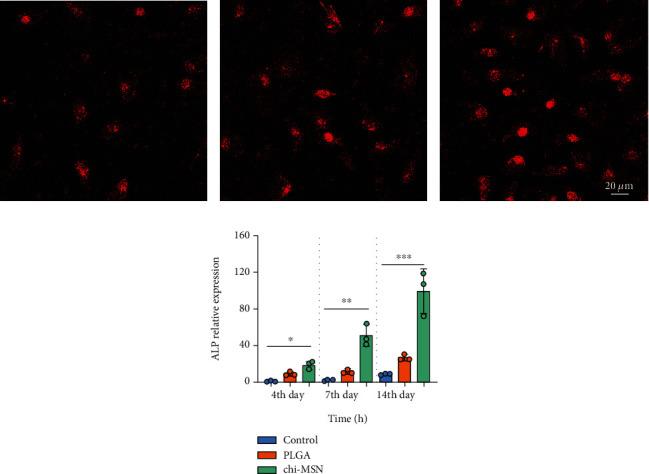
Mineralization of BMSCs. (a–c) ALP fluorescent staining on the 14th day in control, PLGA, and Chi-MSN groups. (d) Quantitative evaluation of ALP expression in control, PLGA, and Chi-MSN groups. ^∗^*P* < 0.05, ^∗∗^*P* < 0.01, and ^∗∗∗^*P* < 0.001.

The mineralization ability of BMSCs is an important index to evaluate the differentiation ability and osteogenic ability of BMSCs [26]. The fluorescence intensity of Chi-MSN-cultured BMSCs was found to be higher compared with control and PLGA groups two weeks after culture (Figures 6(a)–6(c)). According to quantitative analysis, the Chi-MSN group showed an ALP mRNA level 13 times higher than the control group on day 14 after culture (Figure 6(d)). The above results suggest that Chi-MSN can further induce BMSCs to differentiate into osteoblasts and enhance their osteogenic ability.

### 3.6. Chi-MSN Delivery System Effectively Improves the Osteogenic Ability of BMSCs

**Figure 7:**
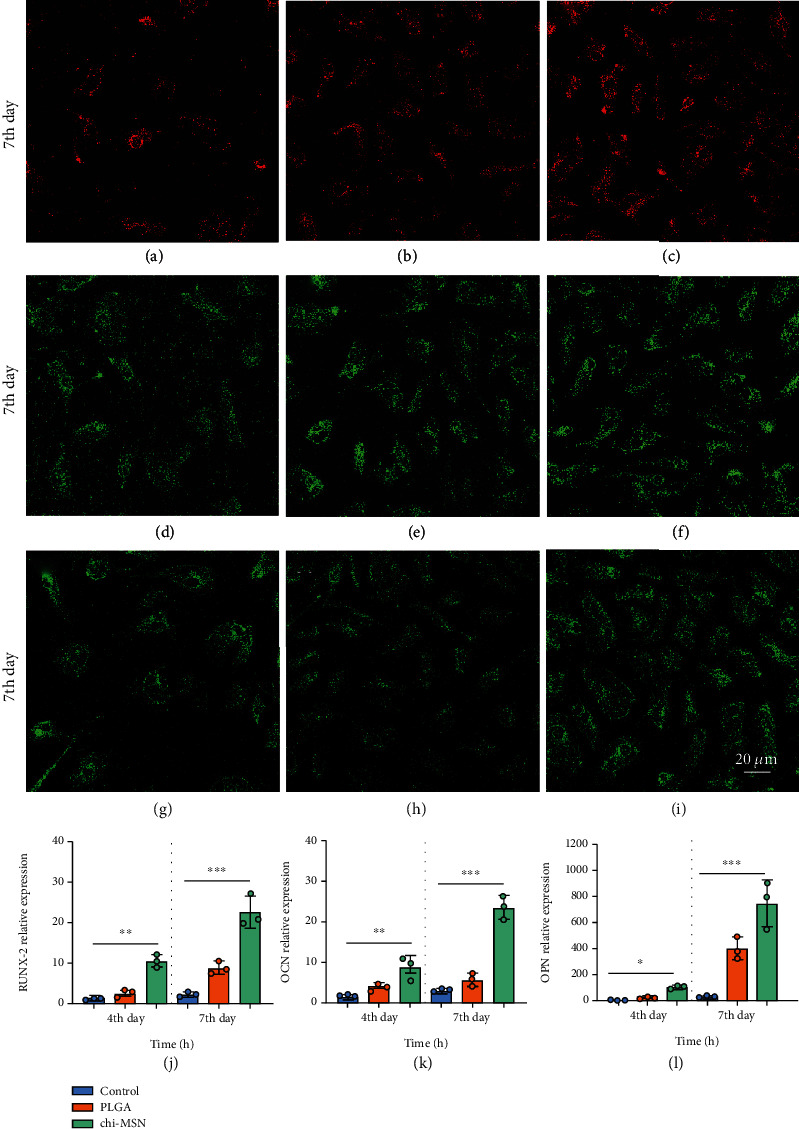
Detection of related osteogenic genes. (a–c) RUNX-2 fluorescent staining results of control, PLGA, and Chi-MSN groups. (d–f) OCN fluorescent staining results of control, PLGA, and Chi-MSN groups. (g–i) OPN fluorescent staining results of control, PLGA, and Chi-MSN groups. (j, k) Quantitative evaluation of RUNX2, OCN, and OPN relative expression in control, PLGA, and Chi-MSN groups. ^∗∗^P < 0.01 and ^∗∗∗^P < 0.001 vs. control or PLGA group.

BMP-2 can activate the expression of specific osteogenic genes through SMAD signal pathway, so as to play the function of bone induction [27]. Therefore, we evaluate the osteogenic ability of Chi-MSN delivery system from related osteogenic gene expression of RUNX2, OCN, and OPN. The fluorescence intensity of Chi-MSN cultured BMSCs was observed to be higher versus control and PLGA groups after two weeks of culture (Figures 7(a)–7(i)). Quantitative analysis showed that RUNX2, OCN, and OPN mRNA relative expressions in the Chi-MSN group were higher (Figure 7(j)–7(k)). The above results indicated that Chi-MSN could better activate BMP-2 signaling pathway, so as to better promote the differentiation and maturation of BMSCs.

### 3.7. Chi-MSN Delivery System Can Better Promote the Repair of Bone Defect

**Figure 8:**
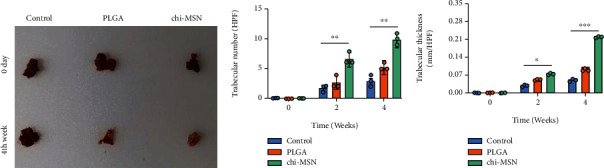
Therapeutic effect of BMP-2@Chi-MSN on bone defect. (a) Macroscopic appearance of lesions in saline, PLGA, or Chi-MSN-treated bone defect mice. (b) Trabecular number of lesions in saline, PLGA, or Chi-MSN-treated bone defect mice. (c) Trabecular thickness of lesions in saline, PLGA, or Chi-MSN-treated bone defect mice. ^∗^*P* < 0.05, ^∗∗^*P* < 0.01, and ^∗∗∗^*P* < 0.001 vs. control or PLGA group.

At last, mouse models of bone defect were established to evaluate the osteogenic capacity of Chi-MSN. As expected, Chi-MSN could better reduce the lesions of bone defect mice, with clean appearance of new bone and no swelling or bleeding, while PLGA could reduce the lesions to a certain extent (Figure 8(a)). Microscopical observation of trabecular number and thickness revealed that the Chi-MSN-treated bone defect mice had higher trabecular number and thicker trabecular than PLGA and saline-treated mice (Figures 8(b) and 8(c)). As a result, Chi-MSN could better promote the repair of bone defect.

## 4. Discussion

With the reform and progress of tissue engineering technology, more and more new technologies are used in the repair of bone defects. BMSCs are often used as seed cells for bone defect repair because of their self-renewal and strong differentiation ability [28]. In order to stimulate the proliferative activity and multidirectional differentiation of BMSCs, related growth factors are often used together with BMSCs, so as to improve the clinical efficacy [29]. BMP-2 belongs to TGF family and is the most widely used bone formation signal protein in biological tissue engineering [30]. It binds to BMP receptor to activate SMAD signal pathway, which will promote related target gene expression, thus forming a complete bone induction pathway in vivo [31, 32]. At the same time, the combined use of Dex can enhance differentiation and bone induction ability of BMSCs and reduce osteoclast activity by inhibiting the release of inflammatory factors, so as to enhance osteogenesis [33, 34]. Therefore, the combination of Dex and BMP-2 provides a new direction for bone defect therapy in CO.

In order to successfully deliver Dex and BMP-2 to the target, we used MSN modified with chitosan as a targeted vehicle. MSN has large internal spatial structure to effectively load Dex and can effectively penetrate the cell membrane [35]. The pH response characteristics of chitosan can effectively reduce Dex loss during transportation in vivo until MSN enters BMSCs [36]. MSN presents favorable and tunable physicochemical properties, mainly manifesting in particle/pore size, pore volume, surface/volume area, pore structure, and surface functionality. Thanks to the porous structure, MSN is able to provide cavities that can accommodate and release biomolecules and therapeutic agents of large quantities [37]. In fact, it is the versatility of MSN in size, morphology, and texture that has driven its application as a nanocarrier for controlled drug delivery [38]. Otherwise, “NH2” of chitosan can form hydrogen bond with BMP-2 to realize noncovalent fixation [39]. Once it arrives at the action target, BMP-2 can be smoothly released from the surface of the delivery system and specifically combined with BMP receptor on the surface of BMSCs to activate the signal pathway [40]. In stability experiment, we found that Chi-MSN delivery system could maintain good stability in vivo, thus effectively reducing drug loss during delivery process. Otherwise, compared with PLGA, Chi-MSN has obvious pH response. In the environment with pH 7.4, less BMP-2 and Dex were released, while at pH 6, the release efficiency was significantly increased, especially Dex. Therefore, Chi-MSN can further reduce drug loss during transportation in vivo and improve the utilization rate of drugs. In terms of permeability, the efficiency of Chi-MSN drug delivery system was significantly higher than that of PLGA, which can deliver more Dex into BMSCs, so as to better improve their activity repair of the defective bone tissue. Therefore, the proliferation activity of BMSCs in the Chi-MSN group was statistically increased versus the PLGA group, with fewer apoptotic cells found at 7th and 14th day after culture.

The mineralization and osteogenic ability of BMSCs are important indexes to evaluate the repair of bone defects. In this study, we selected ALP as the evaluation index of BMSC mineralization ability. It was found that ALP was markedly higher in the Chi-MSN group than in the PLGA group, suggesting that the maturity of osteoblasts was relatively higher. In addition, we discussed the expression of specific osteoblast genes related to BMP-2 signaling pathway, such as RUNX-2, OPN, and OCN. The results showed that the expressions of above indexes were higher in the Chi-MSN group, which further suggested that osteoblasts differentiated more mature and could repair bone defects better. To further evaluate the efficacy of BMP-2@Chi-MSN in repairing bone defects, we conducted animal experiments in the bone defect mouse model. The study found that the number and thickness of bone trabecula in the femoral defect increased more significantly in the Chi-MSN group, which may explain effective and better repair of the bone defect in the focus. Therefore, Chi-MSN delivery system can better deliver BMP-2 and DEX to the target, which is conducive to BMP-2 activating the SMAD signal transduction pathway of BMSCs; otherwise, Dex can better promote the differentiation of BMSCs into osteoblasts. However, there are some limitations in this study, in the experiment, we mainly studied the repair effect of Chi-MSN loaded with BMP-2 on bone defects but did not study the loading of antibiotics. At the same time, we did not conduct in-depth discussion on the scaffold materials for repairing bone defects, which warrants further investigation in the follow-up research.

## 5. Conclusion

We found that Chi-MSN delivery system had good stability and pH responsiveness, which can effectively reduce drug loss during delivery, so as to better deliver BMP-2 and Dex to the target. In BMSC experiment, we found that Chi-MSN could better penetrate into cells, so as to better improve cell activity and reduce apoptosis. Otherwise, after the treatment of Chi-MSN delivery system, BMSCs could better differentiate into osteoblasts, and their mineralization and osteogenic ability could be significantly improved, so as to effectively repair the defective bone tissue. In mouse experiment, we found that in the Chi-MSN group, the number and thickness of bone trabecula in the defective bone tissue were significantly improved, so that the defective bone tissue could be repaired better. Therefore, BMP-2@Chi-MSN delivery system may be a good choice for bone defect repair of CO in the future.

## Figures and Tables

**Table 1:** Primer information of PCR.

	F primer	R primer
RUNX-2	5′-CGGCATGAGAGCACCTTGAC-3′	5′-CTGGCAACCATTACGGAGTC-3′
OCN	5′-CAGGGCAGTAACTTATCTTG-3′	5′-CCTGAACCAAGCCTTACTCA-3′
OPN	5′-ACAGCAACGGGAAGACCAGC-3′	5′-GCTTTGGAACTCGCCTGACTG-3′
GAPDH	5′-GGAGCGAGATCCCTCCAAAAT-3′	5′-GGCTGTTGTCATACTTCTCATGG-3′

## Data Availability

The labeled dataset used to support the findings of this study are available from the corresponding author upon request.
